# Comparison of midterm efficacy of Kirschner wires and elastic intramedullary nails after closed reduction of Judet type 3 radial neck fractures in children: a multicenter study

**DOI:** 10.3389/fped.2024.1350993

**Published:** 2024-02-08

**Authors:** Zheng Xu, Jun Teng, Yuyuan Wu, Feng Xiang, Yuyin Xie, Junqiao Xiang, Can Liu, Zhenqi Song, Zhongwen Tang, Jie Wen, Yanjun Li, Sheng Xiao

**Affiliations:** ^1^ Department of Pediatric Orthopedics, Hunan Provincial People’s Hospital, the First Affiliated Hospital of Hunan Normal University, Changsha, Hunan, China; ^2^ Department of Pediatric Orthopedics, Zhangjiajie People’s Hospital, Zhangjiajie, Hunan, China; ^3^ Department of Pediatric Orthopedics, Traditional Chinese Medicine Hospital in Huaihua, Huaihua, Hunan, China; ^4^ Department of Orthopedics, Zhuzhou Hospital Affiliated to Xiangya School of Medicine, Central South University, Zhuzhou, Hunan, China; ^5^ Department of Anatomy, Hunan Normal University School of Medicine, Changsha, Hunan, China

**Keywords:** Kirschner wires, elastic intramedullary nails, radial neck fractures, Judet classification, Judet type 3

## Abstract

**Objective:**

The objective of this study was to compare the midterm efficacy of Kirschner wires and elastic intramedullary nails after the closed reduction treatment of Judet 3 radial neck fractures in children.

**Methods:**

This was a retrospective multicenter study of patients diagnosed with Judet type 3 radial neck fractures who underwent closed reduction and internal fixation at four tertiary hospitals from January 2019 to December 2021. Gender, age, fracture type, operation time, follow-up time, x-ray results and complications were collected. The recovery of elbow joint between the two internal fixation methods, elbow motion and complications at the last follow-up were compared.

**Results:**

The average operation time of EIN group was statistical significantly increased compared with KW group. There were no significant differences in MEPS score and ROM 3 months after surgery between the two groups, but the ROR Angle of EIN group was statistical significantly increased compared with KW group 3 months after surgery. There were no significant differences in MEPS score, ROM and ROR at the last follow-up. The incidence of complications in EIN group was significantly lower than that in KW group.

**Conclusion:**

The use of elastic intramedullary nails fixation or Kirschner wires fixation in the treatment of radial neck fractures in children can both achieve satisfactory fracture reduction and healing. Compared with elastic intramedullary nails, the operation time of Kirschner wires fixation is shorter, and the internal fixation does not need to be removed under anesthesia again, but the complication rate is higher.

## Introduction

Radial neck fractures account for 5%–10% of elbow fractures in children and usually occur between 8 and 11 years of age (1). The treatment of radial neck fractures in children is challenging, and misdiagnosis or delayed treatment may result in malunion or non-union of the radial neck, resulting in limited elbow movement (2). Judet classification is a commonly used clinical classification method for children with radial neck fractures (3), which is divided into five types according to the Angle between the vertical line at both ends of the radial head fracture and the longitudinal axis of the radial axis. Type 3 fracture defines as displacement >1/2 of transverse diameter and angulation between 30° and 60° (4). The incidence of type 3 fracture counts 41.6% as the most common fracture type, with a high risk of unsatisfactory functional results 11.9% (5). Metaizeau first introduced reduction methods for radial neck fractures and fixed by elastic intramedullary nail (6). Most literature reports are based on closed reduction methods (7, 8), and open reduction is generally regarded as the last resort after the failure of closed reduction techniques, but literature reports (9, 10) a high rate of open reduction, which may lead to catastrophic clinical results, and are more likely to cause complications such as avascular necrosis, premature closure of epiphysis and ectopic ossification (11). Kirschner wires and elastic intramedullary nails are two commonly used internal fixation techniques for children with radial neck fractures, but as there is no clear difference on indications, there is currently few consensus on the optimal internal fixation method for closed reduction. The objective of this study was to compare the midterm efficacy of two different internal fixation methods of closed reduction Kirschner wires and elastic intramedullary nails in the treatment of Judet 3 radial neck fractures in children.

## Methods

We retrospectively reviewed patients diagnosed with Judet type 3 radial neck fractures who underwent closed reduction internal fixation in four tertiary hospitals in Hunan province China between January 2019 and December 2021. All patient data were obtained with the consent of patients and their families before the study, and was approved by the multicenter study ethical commitee of Hunan Provincial People's Hospital(Approval No.20230249).

Inclusion criteria: (1) diagnosis of Judet 3 radial neck fracture; (2) Under 18 years of age; (3) Underwent closed reduction; (4) Elastic intramedullary nails or Kirschner wires internal fixation was used for treatment; (5) No vascular injury; (6) Completely follow-up data.

Exclusion criteria: (1) Judet type I, II or Ⅳ radius neck fracture; (2) Pathological fracture; (3) Open fracture; (4) Fractures accompanied by vascular injury.

Patients who underwent Kirschner wires fixation were included in the KW group, and those who underwent elastic intramedullary nails fixation were included in the EIN group. Information was collected on the child, including sex, age, fracture type, comorbidities, time of operation, follow-up time, x-rays, and complications. Elbow function (MEPS), flexion and extension range of elbow movement (ROM) and range of elbow rotation (ROR) were compared 3 months after treatment and at the last follow-up, and complications were recorded.

### Surgical technique

After general anesthesia, the patient was placed in a supine position with the affected limb at 90 degrees of abduction. Close reduction was attempted. After the elbow should be flexed to 90 degrees for the manipulation, surgeon stabilization of the proximal fragment with the thumb anteriorly while rotating the forearm into full pronation to reduce the shaft to the proximal fragment. If manual reduction is not successful, the end of the Kirchner wire is inserted at the fracture for prying reduction, and fluoroscopy confirms that the radial head is satisfactorily reduced.

In the KW group, a Kirschner wire with a diameter of 1–1.5 mm was used to confirm that the entry point was as close to the proximal end as possible under fluoroscopy to prevent damage to the dorsal interosseous nerve. Through the lateral proximal end of the head of the radius, through the epiphyseal plate and fracture line to the medial end of the metaphysis, the tail of Kirschner wire was left outside the skin.

In the EIN group, a small lateral incision was made 1–2 mm proximal to the growth plate of the distal radius to avoid injury to the cutaneous branch of radial nerve. Drill through the cortex and insert a pre-curved 1.5–2 mm intramedullary nail. Hammer the tail of the nail to hold the intramedullary nail close to the head of the radius, check the forearm pronation and supination angles, and check for stable fixation.

The injured limb was fixed for 4 weeks, and after confirming callus formation, the plaster was removed and rehabilitation training was carried out. In the KW group, Kirschner wire was removed after the plaster removed, and the patient was re-examined every 3 months in the follow up. In the EIN group, the nail was removed from 9 to 12 months after surgery.

### Measurement of outcome

The operation records were thoroughly reviewed, and operation time were recorded. Measurements for the range of elbow movement in fexion/extension(ROM), range of rotation(ROR), Mayo elbow function score (MEPS) were taken at three months post-operation and during the last follow-up (Table 1). The MEPS scale was completed by patients along with their parents’ assistance. Any complications that arose during the follow-up period were duly recorded.

**Table 1:** General information of patients included.

No	Age (yo)	Gender	Group	Side	FU (m)	OP (min)	Complications	3MPost-OP			LFU post-OP		
								MEPS	ROM	ROR	MEPS	ROM	ROR
1	4	F	EIN	R	40	88	N	85	115	90	95	130	135
2	8	M	EIN	L	28	96	N	80	110	85	90	120	140
3	9	M	EIN	L	33	98	Y	75	100	90	85	120	135
4	7	M	EIN	L	36	105	N	75	105	80	90	120	125
5	9	M	EIN	R	32	96	N	85	110	85	90	125	145
6	10	M	EIN	R	26	115	N	85	105	80	90	120	140
7	12	F	EIN	L	38	98	N	80	105	80	85	130	140
8	15	M	EIN	R	29	101	N	85	110	90	90	130	135
9	8	M	EIN	R	41	99	N	80	105	90	95	125	135
10	8	F	EIN	L	40	110	N	75	95	80	90	115	120
11	8	F	EIN	R	31	120	N	85	110	85	90	120	145
12	10	M	EIN	L	34	105	Y	85	105	85	90	120	140
13	7	M	EIN	R	28	90	N	75	105	75	85	115	120
14	6	M	EIN	R	29	110	N	80	115	80	90	125	135
15	9	F	EIN	R	27	100	N	80	110	90	95	130	135
16	6	F	EIN	R	28	116	N	85	110	90	95	120	135
17	8	F	EIN	L	31	120	N	80	105	90	90	130	140
18	11	F	EIN	R	30	110	N	80	105	90	85	125	130
19	6	M	EIN	L	35	105	Y	85	110	85	95	130	125
20	7	M	EIN	R	36	115	N	85	110	90	90	120	130
21	6	M	EIN	L	30	104	N	80	100	90	85	125	130
22	13	F	EIN	L	29	108	N	85	110	90	90	130	135
23	8	F	EIN	R	28	128	N	80	100	90	85	115	135
24	6	M	EIN	L	27	104	N	75	95	75	85	115	125
25	6	F	KW	R	28	69	N	85	105	80	90	125	140
26	8	M	KW	L	30	84	Y	80	100	75	95	120	135
27	7	F	KW	L	34	80	N	85	110	80	90	125	140
28	9	F	KW	R	26	70	Y	75	105	75	85	115	130
29	10	M	KW	R	33	58	Y	80	105	80	85	120	140
30	6	M	KW	R	31	82	N	85	115	75	90	120	135
31	10	M	KW	R	42	65	N	80	120	80	85	125	140
32	7	F	KW	L	38	68	Y	80	115	70	80	120	135
33	9	M	KW	L	26	72	Y	85	125	80	90	130	140
34	16	F	KW	L	27	90	N	70	90	65	80	115	135
35	5	M	KW	L	29	80	N	75	115	75	95	125	140
36	7	M	KW	L	34	80	N	85	120	90	90	130	145
37	9	M	KW	R	39	60	N	80	105	75	90	115	140
38	7	M	KW	R	40	65	N	80	110	80	90	120	135
39	8	F	KW	R	32	65	N	80	120	90	90	125	135
40	9	F	KW	L	37	75	N	85	120	80	90	125	135
41	8	M	KW	R	34	79	N	75	110	75	85	120	145
42	13	M	KW	R	34	86	Y	75	110	75	95	120	135
43	8	M	KW	R	31	60	N	75	100	85	90	110	125
44	13	F	KW	L	29	80	Y	70	95	70	80	110	130
45	6	M	KW	R	34	65	Y	85	115	80	95	120	135
46	4	M	KW	L	35	70	Y	80	110	80	95	120	140

### Statistical analysis

Statistical analysis was performed using SPSS 21.0. The operation time, MEPS score, ROM, ROR were presented as the mean ± SD. All measured data from three months post-operation and the last follow-up were compared using the Mann–Whitney rank sum test. A *P*-value less than 0.05 was considered statistically significant.

## Results

A total of 46 children (28 boys and 18 girls) met the inclusion criteria. Their average age was 8.4 years old (range: 4–16). Radial nerve injury occurred in 11 patients. Of the 46 injuries, 32 were caused by falls and 14 were caused by traffic accidents. Twenty-two patients were fixed by K-wire and 24 by elastic intramedullary nail. There was no significant difference in gender, age, course of disease and fracture type between the two groups (*p* > 0.05).

**Figure 1:**
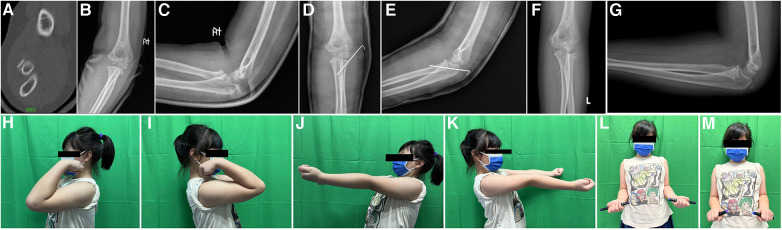
Case No.12, 7 years old girl, diagnosed as radial neck fracture Judet type 3 (**A**–**C**), treated with Kirschner wire (**D**,**E**), after 38 months FU (**F**,**G**), the movement of elbow return normal (**H**–**N**), but this patient had complication of pin tract infection, but it was controlled after oral antibiotics intake.

**Figure 2:**
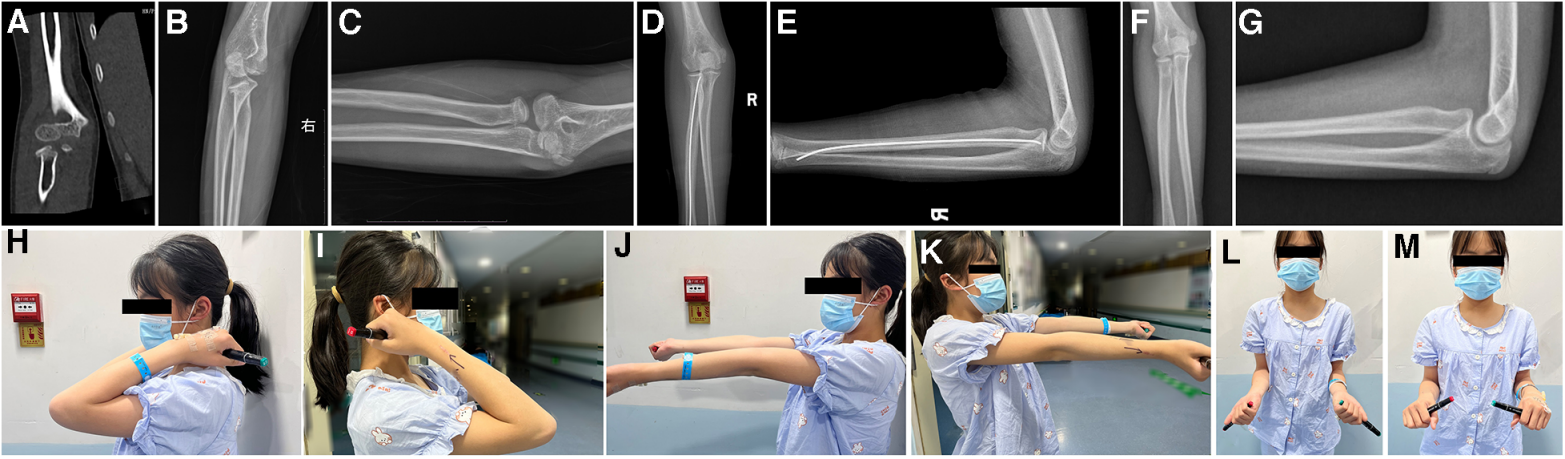
Case No.33, 11 years old girl, diagnosed as radial neck fracture Judet type 3 (**A**–**C**), treated with elastic intramedullary nail (**D**,**E**), after 30 months FU and nails removal (**F**,**G**), the movement of elbow return normal (**H**–**N**).

The mean follow-up time of all children was 32.4 months, including 32.9 months in the KW group and 31.9 months in the EIN group (*P* > 0.05). The mean surgical time was 72.9 min in the KW group and 105.9 min in the EIN group (Table 1). Compared with KW group, operation time in EIN group was statistical significantly increased (*P* < 0.05). In the KW group, the mean MEPS score was 79.5, the mean ROM Angle was 110.0 degrees, and the mean ROR Angle was 78.0 degrees in 3 months follow up post-operation. The mean MEPS score of the EIN group was 81.0, the mean ROM Angle was 106.3 degrees, and the mean ROR Angle was 85.6 degrees. There were no statistic significant differences in MEPS score and ROM in 3 months follow up post-operation between the two groups (*P* > 0.05), but the ROR Angle of EIN group was statistical significantly increased compared with KW group in 3 months follow up post-operation (*P* < 0.05). At the last follow up, the mean MEPS score of the KW group was 88.9 points, the mean ROM Angle was 120.7 degrees, and the mean ROR Angle was 136.8 degrees. The mean MEPS score of the EIN group was 89.6, the mean ROM Angle was 123.1 degrees, and the mean ROR Angle was 133.8 degrees. There were no significant differences in MEPS score, ROM and ROR at the last follow-up between the two groups (*P* > 0.05). The complication rate of the KW group was 40.9%(9/22), including 5 cases of pin tract infection, which was controlled after dress and antibiotics, 2 cases of aggravated postoperative radial nerve injury symptoms, and the neurological symptoms disappeared after neurotrophic therapy, and 2 cases of plaster pressure ulcer complications, which were improved after dress (Figure 1). The complication rate of EIN group was 12.5%(3/24), which was significantly lower than that of KW group (*P* < 0.05). Among them, the symptoms of postoperative radial nerve injury were aggravated in 2 cases and disappeared after neurotrophic therapy (Figure 2), and the complications of plaster pressure sore in 1 case were improved after dress (Table 2).

**Table 2:** Compare of KW group and EIN group.

	KW group	EIN group	*P* value
Total	22	24	
Gender			0.713
Male	14	14	
Female	8	10	
Side			0.979
Left	10	11	
Right	12	13	
Age	8.4	8.4	0.982
FU	32.9	31.9	0.434
OP	72.9	105.9*	0.000
3M PO			
MEPS	79.6	81.0	0.322
ROM	110.0	106.3	0.073
ROR	78.0	85.6*	0.000
LFU PO			
MEPS	88.9	89.6	0.768
ROM	120.7	123.1	0.185
ROR	136.8	133.8	0.124
Complications			
Infections	5	0	
Delayed nerve palsy	2	2	
Cast	2	1	
Complications rate	40.9%	12.5%*	0.028

* *P* < 0.05.

## Discussion

Radial neck fracture is the most common elbow fracture in children, second only to supracondylar fracture of humerus and lateral condylar fracture of humerus, accounting for 5%–10% of elbow fractures in children. The average annual incidence of all elbow fractures in patients under 16 years of age is 12/10,000, among which 14% are radial neck fractures, which are easy to occur in 8–11 years old children (12).

Due to the unique characteristics and different clinical manifestations of bones in children, and unclear expression of their symptoms, fractures are easy to be ignored (13). Therefore, when evaluating a child with a suspected radial neck fracture, the wrist, shoulder, and contralateral upper limb should be thoroughly examined, and the diagnosis should be made based on history, physical examination, and imaging findings. Most of the children have a history of trauma, such as falls, falls or car accidents. In particular, non-displaced radial neck fractures are difficult to detect on initial radiographs, and anterolateral radiographs often do not fully reflect the completeness and authenticity of the fracture, oblique radiographs should be included if necessary. To avoid misdiagnosis, film should be taken with the elbow fully extended as much as possible (14, 15).

The most common injury mechanism for radial neck fractures is forearm supination, falling while extending the arm, and the associated eversion thrust, which causes the lateral head of the radius to strike the head of the humerus, breaking the neck at its weakest point. Symptoms present as pain and limited range of motion. Patients always refuse to move their elbows. A physical examination revealed elbow swelling and pain exacerbated by exercise, especially when attempting pronation, supination, and flexion. The tenderness is mainly localized to the lateral elbow joint (16, 17). In addition, neurovascular examination should be performed, especially the posterior interosseous nerve (18, 19). Attention should also be paid to soft tissue swelling. Forearm compartment syndrome, although rare in radial neck fractures, has serious consequences (20).

In the treatment of radial neck fracture in children, the normal anatomical structure of the proximal radius should be restored first, angulation and varus deformity of the radius head should be corrected to maintain the stability of the elbow joint. Based on this, minimally invasive treatment is indicated to reduce the risk of related complications and the possibility of corresponding iatrogenic injury. The key factors in determining treatment are fracture displacement, fracture Angle, and patient age. Angulation of less than 30° is acceptable because of the potential for bone remodeling to correct the fracture Angle as the child grows. Thus, fractures of the radial neck at angles less than 30° can be treated with closed reduction and plaster immobilization. Displaced radial neck fractures at angles greater than 30° (Judet 3 and IV) should be treated surgically. Kashayi-Chowdojirao et al. (21) suggest that if the child is very young, the fracture Angle <45° can be treated conservatively. In clinical practice, Metaizeau technique and K-wire fixation are both commonly used. Yang et al. proposed that the more severe the fracture displacement, the larger the treatment incision, and the worse the treatment result (5). More invasive treatment methods should be gradually adopted only when minimally invasive methods fail, and open reduction should be considered only when the fracture cannot be reduced to within the displacement range of <30° and <50% displacement (8). Therefore, closed reduction and internal fixation is still the main method for the clinical treatment of radial neck fractures in children.

However, Falciglia et al. (22) suggested that immobilization is not always necessary if the radial head is stable after surgery, but Langenberg et al. (23) concluded that non-fixed fractures have a higher percentage of loss of range of motion after surgery. If the radius neck fracture Angle is greater than 60°, the use of Kirschner wire fixation after closed reduction may have an advantage over elastic intramedullary nail fixation, but this difference is not significant in fractures angled at 31°–60°. In addition, the plaster fixation time of the Kirschner pin group (4–6 weeks) was slightly longer than that of the elastic intramedullary nail group (3–4 weeks). This is because early fixation loss may cause nonunion in children with radial neck fractures (24). In the EIN group, the intramedullary nailing can still stabilize the fracture after the plaster is removed. However, in the KW group, because the cast and Kirschner wire are removed at the same time, a longer cast fixation time may be required after the Kirschner wire is removed to prevent the occurrence of bone nonunion after the cast is removed. Studies have suggested that multiple attempts at closed reduction can lead to muscle stiffness, bleeding, and additional joint damage, which was not the case in our study.

At the same time, we found that patients with fair or poor clinical outcomes were all over the age of 10. As reported by Kumar et al. (8), older than 10 years may be one of the factors associated with a poorer prognosis, and older children tend to suffer more severe fractures and have a poorer prognosis, possibly because older children have higher energy at the time of injury. In addition, the bones of young children have more cartilage and are better cushioned. The energy from the trauma is absorbed more efficiently, and fractures are less severe than in older children, bone also has greater remodeling potential, and therefore can achieve better clinical outcomes. In general, both our study and the literature of other scholars believe that the efficacy of closed reduction and closed reduction in the treatment of various types of radial neck fractures in children is similar, and both Kirschner wire and elastic stabilized intramedullary nail can achieve good clinical results (25).

Various associated elbow injuries occur in 30%–50% of radial neck fractures. The most common concomitant fractures include olecranon fractures, proximal ulna fractures, medial epicondylar fractures, and lateral epicondylar fractures. It has been reported (20) that associated fractures have a negative impact on the functional outcome of radial neck fractures. In our study, out of five patients with associated fractures, three patients had excellent outcomes, one patient had a good outcome, and one patient had a poor outcome. Complications occur in 20%–60% of cases after radial neck fracture in children (26). The most common complications include loss of motion, pain, periarticular ossification, pin tract infection, cubital valgus, nerve palsy, elbow stiffness, radioulnar adhesion, avascular necrosis, posterior interosseous nerve injury, ectopic ossification (27), nonunion, and malunion (12, 14, 21). Ruptures of the medial collateral ligament and accompanying dislocation of the elbow joint are also not uncommon. In our study, the higher complication rate in the Kirschner group may associate with the intra-articular fixation. The K-wire go through elbow joint, one end drill into the articular, another end keep outside of skin, which may lead an infection. The intramedullary nails go through the medullary cavity and did not contact articular directly, which may decrease the infection rate. The path of nails is isolated from nerve, but the path of K-wire may come across nerve, which brings higher risk of nerve injury.

Limitations of this study were the short follow-up time. Therefore, long-term complications such as ischemic necrosis of the radial head and cubital valgus could not be evaluated. Larger sample sizes, randomization, and longer follow-up times are needed for more comprehensive comparisons.

## Conclusions

In summary, both internal fixation methods for children with radial neck fractures can achieve good interim clinical results, and there is no significant difference in clinical effect. Compared with elastic intramedullary nails, the operation time of Kirschner wire fixation is shorter, and the internal fixation does not need to be removed by anesthesia again, but the complication rate is higher than that of elastic intramedullary nails. Early use of antibiotics in the Kirschner wire group can effectively prevent the complication of pin infection.

## Data Availability

The original contributions presented in the study are included in the article/Supplementary Material, further inquiries can be directed to the corresponding authors.
